# Chemical composition and cytotoxic properties of *Clinacanthus nutans* root extracts

**DOI:** 10.1080/13880209.2016.1242145

**Published:** 2016-12-08

**Authors:** Peik Lin Teoh, Angelina Ying Fang Cheng, Monica Liau, Fui Fui Lem, Grace P. Kaling, Fern Nie Chua, Bo Eng Cheong

**Affiliations:** a Biotechnology Research Institute, Universiti Malaysia Sabah, Jalan UMS, Kota Kinabalu, Sabah, Malaysia;; b Faculty of Science & Natural Resources, Universiti Malaysia Sabah, Jalan UMS, Kota Kinabalu, Sabah, Malaysia

**Keywords:** *Clinacanthus nutans*, anti-proliferative, apoptosis, BAX, BCL2

## Abstract

**Context:***Clinacanthus nutans* Lindau (Acanthaceae) is a medicinal plant that has been reported to have anti-inflammatory, antiviral, antimicrobial and antivenom activities. In Malaysia, it has been widely claimed to be effective in various cancer treatments but scientific evidence is lacking.

**Objective:** This study investigates the chemical constituents, anti-proliferative, and apoptotic properties of *C. nutans* root extracts.

**Materials and methods:** The roots were subjected to solvent extraction using methanol and ethyl acetate. The anti-proliferative effects of root extracts were tested at the concentrations of 10 to 50 μg/mL on MCF-7 and HeLa by using 3-(4,5-dimethylthiazol-2-yl)-2,5-diphenyl tetrazolium bromide (MTT) assay for 72 h. Morphological changes were observed under light microscope. Pro-apoptotic effects of root extracts were examined using flow cytometric analysis and RT-PCR. The chemical compositions of root extracts were detected using GC-MS.

**Results:** The proliferation of MCF-7 cells was inhibited with the IC_50_ values of 35 and 30 μg/mL, respectively, for methanol and ethyl acetate root extracts. The average inhibition of HeLa cells was ∼25%. Induction of apoptosis in MCF-7 was supported by chromatin condensation, down-regulation of *BCL2* and unaltered expression of *BAX*. However, only ethyl acetate extract caused the loss of mitochondrial membrane potential. GC-MS analysis revealed the roots extracts were rich with terpenoids and phytosterols.

**Discussion and conclusions:** The results demonstrated that root extracts promote apoptosis by suppressing *BCL2* via mitochondria-dependent or independent manner. The identified compounds might work solely or cooperatively in regulating apoptosis. However, further studies are required to address this.

## Introduction

Despite the advancement in technology, breast cancer remains the most common occurring cancer and the leading cause of cancer death in women worldwide. It has contributed to worldwide 23% and 14% of total new cancer cases and deaths in 2008 (Jemal et al. 2011). The conventional treatments for breast cancer include surgery, radiation, chemotherapy and hormonal therapy. However, their efficacy is still unsatisfactory and prolonged use of drugs may render therapeutic resistance (Coley 2008). Resistance to the apoptotic induction is one of the multiple mechanisms evolved in cancer cells. Tremendous research has been focused on finding natural compounds that can modulate apoptosis pathway for novel drug development. Hence, various traditional plants with known medicinal properties have been widely studied in the past decades.

Apoptosis is a physiological mechanism of cell death which genetically programmed cells to undergo suicide. Apoptosis causes morphological features which can be characterized by cytoplasmic blebbing, chromatin condensation, cell shrinkage and nuclear fragmentation (Kiechle & Zhang 2002). Apoptosis pathway is composed of upstream regulators and downstream effector components (Adams & Cory 2007). It can occur via extrinsic or intrinsic pathway. The activation of the intrinsic pathway is regulated by BCL2 family proteins. They can be divided into pro-apoptotic proteins that promote apoptosis such as BAX; and anti-apoptotic proteins such as BCL2 (Gupta 2003). BCL2 arrests apoptosis by inhibiting the release of cytochrome c from mitochondria which directly inactivate caspase 3. BAX acts in an opposite manner and promotes apoptosis by facilitating the release of cytochrome c from mitochondria and thereby triggering caspase activity. Apoptosis induction is recognized as an active strategy to arrest proliferation of cancer cells (Gupta 2003). Hence, the ability of natural compound to induce apoptosis without aggravating other normal cells is therefore the key target in chemoprevention and chemotherapeutic intervention (Fulda 2010; Kuno et al. 2012).

*Clinacanthus nutans* Lindau (Acanthaceae) is a small shrub native to Asian countries (Sakdarat et al. 2009). In Malaysia, it is known as Sabah Snake Grass or Belalai Gajah. In folklore medicine, this plant has been used to treat diabetes mellitus, fever, diarrhoea, and dysuria, when consumed in the form of herbal tea (Uawonggul et al. 2011). In Thailand, the fresh leaves are locally used for the treatment of herpes simplex skin infection, shingles, and recommended for relieving insect bites (Thawarananth 1992; Tuntiwachwuttikul et al. 2004). In addition, this plant also shows antiviral (Jayavasu et al. 1992), immune response (Sriwanthana et al. 1996), anti-inflammatory (Wanikiat et al. 2008), antioxidant (Pannangpetch et al. 2007), and anti-proliferative activities (Yong et al. 2013). It is also effective toward antivaricella-zoster virus infection and recurrent aphthous ulcer (Buajeeb & Kraivaphan 1994; Sangkitporn et al. 1995). In Malaysia, there are emerging testimonies which claim that *C. nutans* is effective in treating cancer (Yong et al. 2013). However, the underlying mode of action is unclear. Furthermore, there is no report on the cytotoxic properties of the plant’s roots as all studies were done using the aerial part of the plants such as leaf and stem. Therefore, this study was carried out to examine the capability of *C. nutans* root extracts in growth inhibition and apoptosis induction of human breast cancer cell line. To narrow down the search of potential bioactive compound(s), we also studied the chemical compositions of the root extracts by using gas chromatography mass spectrometry (GC-MS). The research outcome will furnish our knowledge on the efficacy of using *C. nutans* for chemoprevention or chemotherapy.

## Materials and methods

### Cell lines, media and chemicals

Human breast cancer cell line (MCF-7, HTB-22), human cervical cancer cell line (HeLa, CCL-2) and mouse embryonic fibroblast cell line (NIH 3T3, CRL-1658) were obtained from the American Type Culture Collection (Rockville, MD). Roswell Park Memorial Institute (RPMI) 1640, 10,000 U/mL penicillin/10,000 μg/mL streptomycin, 1x phosphate-buffered saline (PBS) and 2.5 g/L trypsin-1 mmol/L EDTA were obtained from Nacalai Tesque (Kyoto, Japan). Fetal bovine serum (FBS) was purchased from J R Scientific (Woodland, CA). The authentic standards for compounds identification (at least 95% purity), which were squalene, campesterol, stigmasterol, sitosterol, lupeol and betulin were purchased from Sigma-Aldrich (St. Louis, MO). Solvents such as methanol and ethyl acetate (analytical and HPLC grade) were purchased from Fisher Scientific (Leicestershire, UK).

### Preparation of plant extracts

Fresh plant of *C. nutans* was purchased from herbal supplier in Kota Kinabalu, Sabah. The plant was verified by a botanist from the Faculty of Science and Natural Resources, Dr. Berhaman Ahmad. A voucher specimen (ACCN 001/2013) was deposited in the herbarium of Universiti Malaysia Sabah. The roots were thoroughly cleaned, freeze-dried, and the dried roots were then grounded into powder by using a heavy duty blender. Plant powder was soaked in methanol and ethyl acetate with the ratio of one part of powder to ten parts of solvent. The mixtures were placed in a rotary shaker for four days at 25 °C. The mixture was filtered and concentrated under reduced pressure in a rotary evaporator. The extracts were freeze-dried and stored at −80 °C.

### Cell culture

MCF-7, HeLa and NIH 3T3 cell lines were grown in RPMI 1640 media supplemented with 10% FBS and incubated at 37 °C in a humidified atmosphere containing 5% CO_2_.

### Anti-proliferation assay

A seeding density of 1 × 10^4^ cells was grown in 96-well plates and incubated in a CO_2_ incubator at 37 °C for overnight. The cells were treated with root extracts at the concentrations ranging from 10 to 50 μg/mL for up to three days. MTT assay was performed using Cell Proliferation Kit I (Roche Diagnostics, Mannheim, Germany) according to manufacturer’s protocol. The optical density was measured at 550 nm using a microplate reader (Molecular Devices, Sunnyvale, CA). The 50% growth inhibitory concentration (IC_50_) was defined as plant extract’s concentration required for 50% inhibition of cell growth.

### Methylene blue staining

After seeding, cells were treated with root extracts at the IC_50_ values for up to three days. The medium was discarded and cells were washed with PBS for three times. Staining was performed using 0.4% methylene blue for 20 min. Then, methylene blue was removed and cells were washed with PBS for three times. Cell morphological changes were observed under an inverted microscope (Nikon, Japan).

### JC-1 staining

Apoptosis assay was performed using BD^TM^ MitoScreen Kit (BD Biosciences, San Jose, CA) according to manufacturer’s protocol. Briefly, 3 x 10^4^ cells were seeded in 6-well plates and treated with root extracts with the concentrations of 20 to 40 μg/mL for three days. Cells were trypsinized and transferred into a sterile tube. The cells were centrifuged at 400 *g* for 5 min at room temperature. Supernatant was discarded and 500 μL of JC-1 solution was added. Cell was incubated at 37 °C for 15 min. After that, cells were washed twice using 1x assay buffer and centrifuged at 400 *g* for 5 min. Cells were resuspended with 500 μL of 1x assay buffer and analyzed using BD FACSAria flow cytometer (BD Bioscience, San Jose, CA).

### RNA extraction and RT-PCR

Total RNA was extracted using RNeasy kit (Qiagen, Valencia, CA) according to the manufacturer’s protocol. RT-PCR was performed using OneStep RT-PCR kit (Qiagen, Valencia, CA). The components of RT-PCR reaction were RNA template (50 to 100 ng/μL), 0.6 μM gene specific primers, 400 μM dNTPs mix, 1x RT-PCR buffer and enzyme mix. Reverse transcription was carried out at 50 °C for 30 min. The PCR cycling conditions were 95 °C for 15 min, 95 °C for 20 sec, 55 °C to 63 °C for 30 sec, 72 °C for 20 sec, 72 °C for 5 min. The PCR was repeated for 26 to 31 cycles. After completion of PCR, the products were subjected to 2% agarose gel electrophoresis followed by ethidium bromide staining. Primers used in this study were *ACTB* (5′-AGAGCTACGAGCTGCCTGAC-3′,5′-GACATCCGGTTGTGTCACGA-3′), *BAX* (5′-TTTTCGTTCAGGGTTTCATCCA-3′, 5′-TAGAAAAGGGCGACAACCCG-3′) and *BCL2* (5′-GGATAACGGAGGCTGGGATGC-3′, 5′-AACAGCCTGCAGCTTTGTTTC-3′).

### GC-MS analysis

The dried root extracts were dissolved in HPLC grade methanol or ethyl acetate to appropriate concentration. The extract (1 μL) was injected into a GC-MS (GC model 7890, MS model 5975C, Agilent Technologies, Santa Clara, CA) after filtered with 0.22 μm syringe filter. GC separation was performed on a HP-5MS capillary column (Agilent Technologies, Santa Clara, CA) operating at electron impact mode at 70 eV. Pure helium gas with built-in purifier was used at a constant flow rate of 1 mL/min employed in a splitless mode with injector at 250 °C and ion source at 280 °C. The stepped temperature program was as follows: initial temperature oven was started at 220 °C and hold for 5 min and followed by a ramp to 300 °C at 5 °C/min and hold for another 15 min. A post-run of 5 min at 300 °C was sufficient for the next sample injection. Mass analyzer was used in full scan mode scanning from *m/z* 40 to 550 and mass spectra were taken at 70 eV. The identification of compounds was based on the comparison of their mass spectra with standards and also with the library of National Institute Standard and Technology (NIST) version 2.0, with the aid of Automated Mass Spectral Deconvolution and Identification (AMDIS) software version 2.70 by deconvoluting the chromatography peak at the corresponding retention time. Further confirmation of the identity of the chromatographic peaks was done by spiking using reference standards.

## Results

### Anti-proliferative effect of C. nutans root extracts

**Figure 1.**
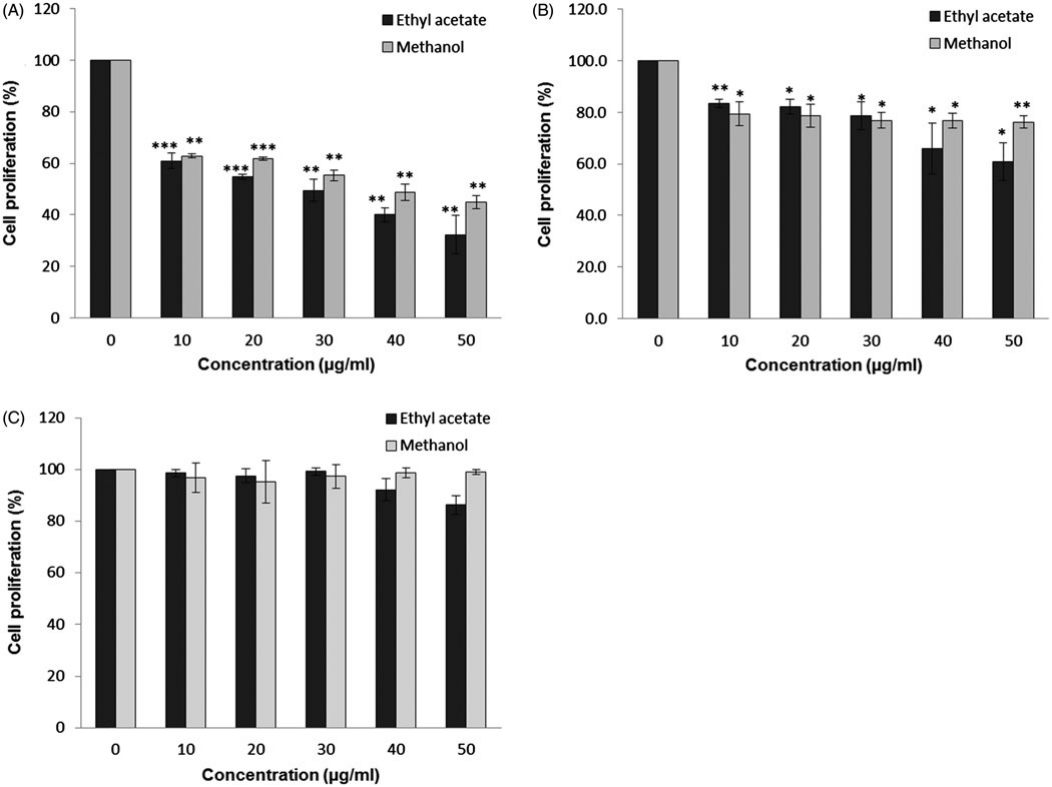
The anti-proliferative effect of *C. nutans* root extracts. Treatments were performed on (a) MCF-7, (b) HeLa and (c) 3T3 cells for three days. Data represent three independent experiments performed in triplicate. Asterisks denote differences with statistical significances compared to untreated cells (*, ** and *** represent *p* < 0.05, *p* < 0.005 and *p* < 0.0005 respectively). *p*-values were obtained from a two-tailed *t*-test.

A significant growth inhibition of MCF-7 and HeLa could be seen at the concentration of 10 μg/mL for both methanol and ethyl acetate root extracts (Figure 1). The growth inhibition of MCF-7 cells was gradually increased when the concentration of both extracts increased (Figure 1(a)). However, the growth inhibition of HeLa cells caused by both root extracts did not reach 50% at the tested concentrations (Figure 1(b)). Both extracts showed no or little inhibition on NIH 3T3 normal cells (Figure 1(c)). From Figure 1(a), the IC_50_ values of MCF-7 cells treated with methanol and ethyl acetate extracts were 35 and 30 μg/mL, respectively. In contrast, the percentage of growth inhibition for HeLa was about 20 to 40% at the concentrations of 40 to 50 μg/mL (Figure 1(b)). This indicates that the cytotoxicity effect of root extracts is selective towards cancer cells and MCF-7 cells are more susceptible to *C. nutans* treatment compared to HeLa cells. Therefore, pro-apoptotic activity of root extracts was focused on MCF-7 cells only.

### Nuclear morphological change

**Figure 2.**
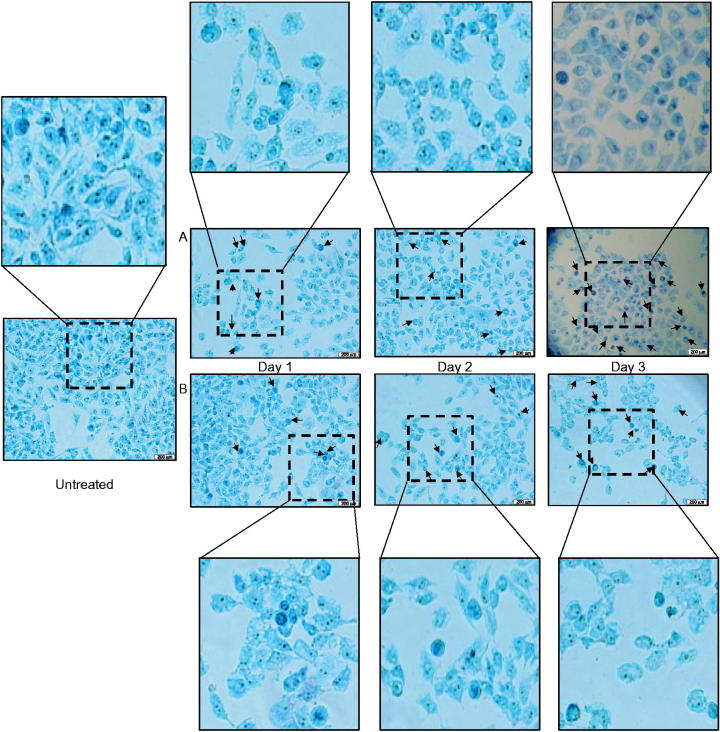
Morphological changes caused by *C. nutans* extracts on MCF-7. Cells were treated with (a) ethyl acetate and (b) methanol root extracts of *C. nutans* at their respective IC_50_ value for up to three days. Cells were stained with methylene blue and observed under microscope with 40x magnification. Arrows indicate some cells with morphological changes when compared with untreated cells.

As shown in Figure 2, MCF-7 cells treated with root extracts began to exhibit peripheral nuclear membrane condensation after one day treatment compared to untreated cells. The occurrence of the morphological changes and nuclear condensation became more profound in the cell population when the duration of the treatment increased to three days. The morphological changes caused by camptothecin were depicted in Figure S1. Comparing to methanol root extracts (Figure 2(b)), ethyl acetate root extract caused more distinct morphological change after three days treatment (Figure 2(a)). Besides, cell shrinkage, loss of cell contact, and chromatin condensation were observed in the treated cells. These suggested that cells might undergo apoptosis after treatment.

### The apoptotic effect of C. nutans root extracts

**Figure 3.**
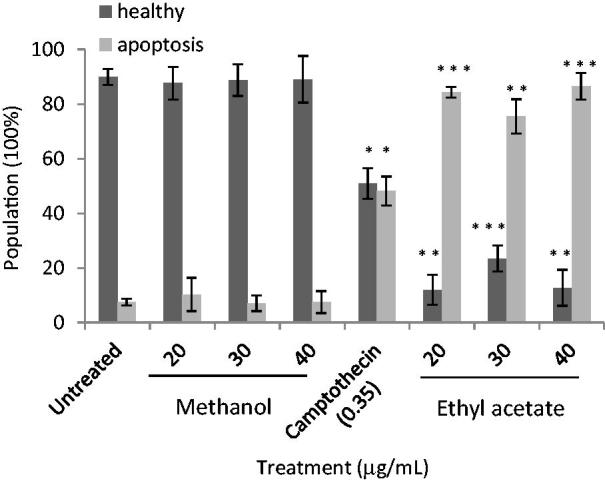
Apoptotic effects of *C. nutans* root extracts on MCF-7 cells. Cells were treated with *C. nutans* root extracts and camptothecin for three days at the indicated concentrations. Data represent three independent experiments performed in triplicate. Asterisks denote differences with statistical significances compared to untreated cells (*, ** and *** represent *p* < 0.05, *p* < 0.005 and *p* < 0.0005 respectively). *p*-values were obtained from a two-tailed *t*-test.

For untreated samples, about 90% of cell populations were in healthy condition but upon camptothecin treatment at the concentration of 0.35 μg/mL, about 50% of cells loss their mitochondrial membrane potential (Figure 3). Surprisingly, loss of mitochondrial membrane potential was higher in cells treated with acetyl acetate extract (∼78 to 82%) than camptothecin. In contrast, integrity of inner mitochondrial membrane was preserved in cells treated with methanol extract as the proportional of healthy and apoptotic cells were similar to untreated cells. Methanol root extract did not exhibit loss of mitochondrial potential as compared to ethyl acetate root extract, suggesting the induction of apoptosis by methanol root extract is mitochondria independent.

**Figure 4.**
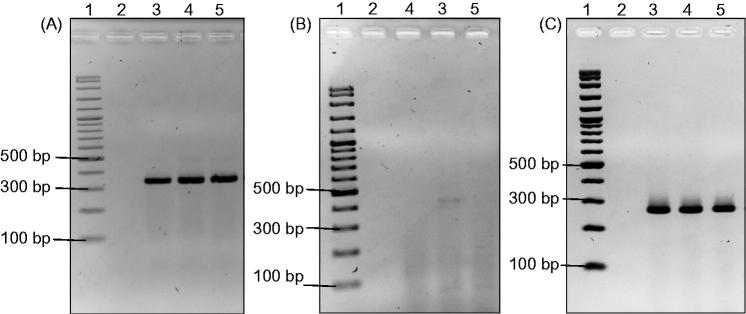
The effect of *C. nutans* root extracts on the expression of *ACTB* (a), *BCL2* (b) and *BAX* (c) in MCF-7 breast cancer cell line by RT-PCR. Lane 1: 100 bp DNA marker, lane 2: negative control, lane 3: without treatment, lane 4: methanol root extract and lane 5: acetyl acetate root extract.

The total RNA of MCF-7 cells treated with methanol and ethyl acetate root extracts were extracted after three days treatment. The effect of root extracts on the expression of *BCL2* and *BAX* genes was evaluated using RT-PCR. *ACTB* is the housekeeping gene used in this study. The expected size of the *ACTB* amplicon was ∼350 bp and its expression level was not altered upon treatments (Figure 4(a)). Based on Figure 4(b), *BCL2* expression was only detected in untreated samples (Lane 3) with the size of ∼450 bp. On the other hand, the expression of *BCL2* in samples treated with both root extracts was undetectable (Lanes 4 & 5). As for the expression of *BAX*, we found no alteration of band intensity (∼290 bp) in both treated and untreated MCF-7 cells (Figure 4(c)). These results indicate that *C. nutans* root extracts promote apoptosis by suppressing the expression of anti-apoptotic gene (*BCL2*) while maintaining the expression of pro-apoptotic gene (*BAX*) in MCF-7 cells.

### Chemical composition of the root extracts of C. nutans

**Figure 5.**
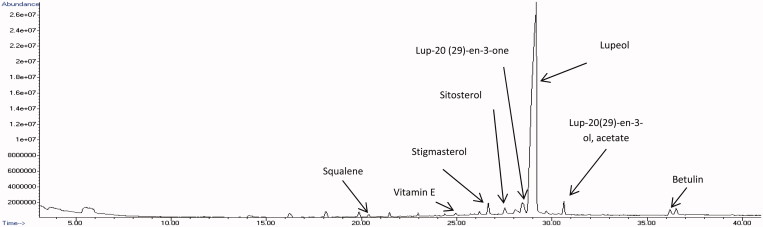
GC-MS chromatogram of ethyl acetate root extract of *C. nutans*.

**Figure 6.**
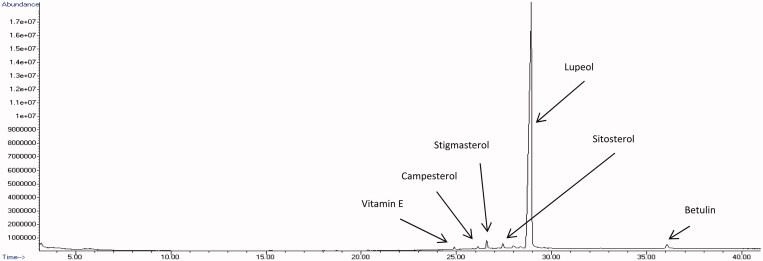
GC-MS chromatogram of methanol root extract of *C. nutans*.

The identified bioactive compounds were characterized according to their retention time, molecular formula, molecular weight, peak area (%) and only those with reported cytotoxic effects on cancer cells were summarized in Table 1 and Table 2. The GC-MS total ion chromatograms of ethyl acetate and methanol root extracts were depicted in Figures 5 and 6, respectively. The results revealed more compounds were found in ethyl acetate root extract (Table 1) compared to methanol root extract (Table 2). The highest amount of compounds found in ethyl acetate root extract (Figure 5) was lupeol (79.05%), followed by lup-20(29)-en-3-one (2.79%), lup-20(29)-en-ol acetate (1.50%), stigmasterol (1.50%), sitosterol (1.15%), β-amyrin (1.15%), betulin (0.96%), campesterol (0.37%), squalene (0.34%), vitamin E (0.34%) and oleic acid (0.22%). Meanwhile, the methanol root extract (Figure 6) consisted of lupeol (94.21%), betulin (1.38%), stigmasterol (1.33%), sitosterol (1.01%), β-amyrin (0.82%), vitamin E (0.39%) and campesterol (0.39%) but not squalene, oleic acid and other lupeol derivatives. However, the most abundant compound found in both root extracts was lupeol.

**Table 1.** Cytotoxic compounds identified in the ethyl acetate root extract of *C. nutans*.

No.	Retention time (min)	Bioactive compound	Nature of compound	Peak area (%)	Molecular formula	Molecular weight	Reported cytotoxic effect on cancer cells
1	3.675	Oleic acid	Fatty acid	0.22	C_18_H_34_O_2_	282.46	Nelson 2005; Menendez et al. 2006
2	20.374	Squalene	Triterpenoid	0.34	C_30_H_50_	410.71	Nakagawa et al.^1985^; Rao et al. 1998; De Los Reyes et al. 2015
3	24.956	Vitamin E	Fat soluble vitamin	0.34	C_29_H_50_O_2_	430.70	Ramdas et al. 2011
4	26.191	Campesterol	Phytosterols	0.37	C_28_H_48_O	400.68	Awad et al. 2003
5	26.658	Stigmasterol	Phytosterols	1.50	C_29_H_48_O	412.69	Ghosh et al. 2011; Lee et al. 2014
6	27.532	gamma-Sitosterol and beta-Sitosterol	Phytosterols	1.15	C_29_H_50_O	414.70	Awad et al. 2003; Chai et al. 2008; Sundarraj et al. 2012
7	28.441	Lup-20(29)-en-3-one	Triterpenoid	1.69	C30H48O	424.70	Hata et al. 2002, 2008
8	28.500	Lup-20(29)-en-3-one	Triterpenoid	1.10	C30H48O	424.70	Hata et al. 2002, 2008
9	28.806	beta-amyrin	Triterpenoid	1.15	C_30_H_50_O	426.71	Lee et al. 2014
10	28.972	Lupeol	Triterpenoid	26.40	C_30_H_50_O	426.71	Saleem 2009; Saleem et al. 2009; Pitchai et al. 2014; Lee et al. 2014
11	29.065	Lupeol	Triterpenoid	19.50	C_30_H_50_O	426.71	Saleem 2009; Saleem et al. 2009; Pitchai et al. 2014; Lee et al. 2014
12	29.182	Lupeol	Triterpenoid	33.15	C_30_H_50_O	426.71	Saleem et al. 2009; Pitchai et al. 2014; Lee et al. 2014
13	30.621	Lup-20(29)-en-3-ol, acetate	Triterpenoid	1.50	C_32_H_52_O_2_	468.75	Lee et al. 2014
14	36.199	Betulin	Triterpenoid	0.96	C_30_H_50_O_2_	442.73	Kommera et al. 2011

**Table 2.** Cytotoxic compounds identified in the methanol root extract of *C. nutans*.

No.	Retention time (min)	Bioactive compound	Nature of compound	Peak area (%)	Molecular formula	Molecular weight	Reported cytotoxic effect on cancer cells
1	24.898	Vitamin E	Fat soluble vitamin	0.39	C_29_H_50_O2	430.70	Ramdas et al. 2011
2	26.139	Campesterol	Phytosterols	0.39	C_28_H_48_O	400.68	Awad et al. 2003
3	26.600	Stigmasterol	Phytosterols	1.33	C_29_H_48_O	412.69	Ghosh et al. 2011; Lee et al. 2014
4	27.445	gamma-Sitosterol and beta-Sitosterol	Phytosterols	1.01	C_29_H_50_O	414.70	Awad et al. 2003; Chai et al. 2008; Sundarraj et al. 2012
5	27.987	beta-amyrin	Triterpenoid	0.82	C_30_H_50_O	426.7174	Lee et al. 2014
6	28.943	Lupeol	Triterpenoid	94.21	C_30_H_50_O	426.71	Saleem 2009; Saleem et al. 2009; Pitchai et al. 2014; Lee et al. 2014
7	36.065	Betulin	Triterpenoid	1.38	C_30_H_50_O_2_	442.73	Kommera et al. 2011

## Discussion

Although the inhibitory effect of *C. nutans* extracts have been observed in several other cell lines, this study is the first report on the cytotoxic and apoptotic effects of *C. nutans* root extracts as previous studies have focused on extracts obtained from other parts of the plant (Yong et al. 2013; Arullappan et al. 2014). As different parts of plant such as leaf, bark, stem and roots may contain different types and concentrations of compounds, the efficacy of plant extracts will be varied as well. Overall, the decrease of cell proliferation in MCF-7 and HeLa cells were observed after treatment. However, its anti-proliferative effect is cell-type dependent as inhibition is more significant in MCF-7 than HeLa. Besides, no deleterious effect was found in normal cells, NIH 3T3. The susceptibility of various cancer cell lines towards different types of extracts from various parts of this plant has been clearly reported by other researchers as well (Yong et al. 2013; Arullappan et al. 2014).

The results have demonstrated that both root extracts were capable of inducing apoptosis as substantiated by morphological changes such as peripheral nuclear condensation and cell shrinkage, as well as suppression of anti-apoptotic gene, *BCL2*. However, the effects on the mitochondrial membrane potential were different when compared to untreated cells. It is suggested that apoptosis induced by methanol root extract is mitochondria independent. Although mitochondrial pathway is governed by BCL-2 family proteins which regulate the release of cytochrome c, mitochondria independent apoptosis has reported before (Godefroy et al. 2004; Sinha et al. 2013). Discrepancy of both root extracts could be due to an alternative mechanism of releasing cytochrome c from mitochondria. It has been reported that this phenomenon can happen when there is functional changes of some ion transporter in the inner mitochondrial membrane (Reed et al. 1998). The increase of mitochondrial permeability through the loss of mitochondrial potential as shown in ethyl acetate root extract treated cells could promote the translocation of pro-apoptotic proteins such as BAX to mitochondria results in the release of apoptogenic proteins such as cytochrome c. This also antagonizes the effect of anti-apoptotic proteins such as BCL2 which is known to prolong cell survival (Davids & Letai 2012; Weyhenmeyer et al. 2012). Besides, similar expression profile of *BAX* and *BCL2* but different effect on mitochondrial potential elicited by both root extracts have also indicated the possible involvement of upstream regulatory proteins such as p53, which has been shown to regulate apoptosis via a mitochondria-independent manner (Godefroy et al. 2004).

Based on GC-MS analysis, the *C. nutans* roots are rich with phytosterols such as stigmasterol, campesterol, β/γ-sitosterol, and triterpenes such as lupeol, β-amyrin and betulin. The detected compounds such as lupeol, stigmasterol and betulin have also been reported previously by other researchers (Dampawan et al. 1977; Lin et al. 1983; Aslam et al. 2015). Compounds previously found in various leaf or stem extracts such as C-glycosyl flavones (vitexin, isovitexin, shaftoside, isomollupentin 7-*O*-β-glucopyranoside, orientin and isoorientin), sulfur-containing glucosides (clinamide A-C, 2-cis entadamide A, entadamide A, entadamide C, *trans*-3-methylsulfinyl-2-propenol), cerebrosides and a monoacylmonogalatosylglycerol are undetectable in this study (Dampawan et al. 1977; Lin et al. 1983; Teshima et al. 1997, 1998; Tuntiwachwuttikul et al. 2004; Sakdarat et al. 2009; Tu et al. 2014; Aslam et al. 2015). However, differences in the extraction techniques, detection methods, and plant materials or parts might jeopardize the extractability and identification of some compounds. Being the most abundant compound found in this study, it is reasonable to suggest that lupeol might play role in modulating apoptosis process. Besides, this has been demonstrated in a number of *in vitro* and *in vivo* studies. For instance, lupeol was found to promote apoptosis by inhibiting Ras signalling pathway in human pancreatic adenocarcinoma cells, while Fas-mediated apoptosis was reported in prostate cancer cells (Saleem et al. 2005a,b). In addition, lupeol exhibited growth inhibition of human metastatic melanoma cells *in vitro* and *in vivo* by inducing apoptosis and G1-S phase cell cycle arrest (Saleem et al. 2008). In another study, lupeol was also shown to induce apoptosis in MCF-7 cells by down-regulating BCL2 and BCL-XL expressions (Pitchai et al. 2014).

Despite the minute amount of other identified compounds, their synergistic biological activities could not be neglected. It is worth mentioning that some of them have been reported to have apoptotic effect. For example, stigmasterol was shown to induce apoptosis in Ehrlich’s ascites carcinoma in mice through the activation of protein phosphatase 2A via ceramide (Ghosh et al. 2011). Meanwhile, γ-sitosterol was found to suppress c-MYC formation while promoting caspase activity in breast and lung cancer cells (Chai et al. 2008; Sundarraj et al. 2012). Betulin-induced apoptosis in colon cancer cells via the induction of caspase activity (Kommera et al. 2011). The low number of cancer incidence was found to be associated with the daily consumption of olive oil in Mediterranean diet. Studies have suggested that oleic acid has specifically repressed the transcriptional activity of *Her-2/neu*, which is commonly overexpressed in breast cancer cells (Menendez et al. 2006). Additionally, a pentacyclic triterpenoid, squalene found in olive oil and liver oil of sharks has demonstrated anti-proliferative activity against HCT-116 and MCF-7 cells with the IC_50_ of 4.21 and IC_50_ 5.92 μg/mL, respectively (De Los Reyes et al. 2015). Another triterpenoid called β-amyrin also showed cytotoxicity activity against MCF-7 cells with the IC_50_ of 15.5 μg/mL (Lee et al. 2014). Besides these published anti-proliferative effects in various cancer cell lines, several purified and semi-purified compounds in our laboratory such as lupeol, squalene and phytosterols (mixture of stigmasterol, β-sitosterol and campesterol) were tested on MCF-7 cells to confirm their cytotoxic effect (unpublished data). Taken together, the apoptotic promoting activity of *C. nutans* root extracts might be associated with the high amount of lupeol and also other minute compounds such as squalene and betulin. Nonetheless, how these compound(s) modulate the underlying mechanisms will need further investigation.

## Conclusions

The findings of this study showed that *C. nutans* root extracts are selective towards cancer cells without affecting the proliferation of normal cell line. Although both root extracts exhibited apoptotic effect based on morphological changes and the suppression of *BCL2* expression. Ethyl acetate root extract promoted mitochondrial-dependent apoptosis in MCF-7 cells but this was not shown in cells treated with methanol root extract. The compounds present in both root extracts might work solely or cooperatively in regulating the apoptosis event.

## Supplementary Material

P.__L._Teoh_et_al_supplemental_content.zip
