# Supplementary material for: Chemical composition and cytotoxic properties of Clinacanthus nutans root extracts
Source: Pharm Biol. 2016 Dec 8;55(1):394–401. doi: 10.1080/13880209.2016.1242145 (PMC6130766; doi:10.1080/13880209.2016.1242145)
Supplement: P.__L._Teoh_et_al_supplemental_content.zip [file IPHB_A_1242145_SM1219.zip › P. L. Teoh et al supplemental content.docx]

**200 µm**

200 µm

**200 µm**

200 µm


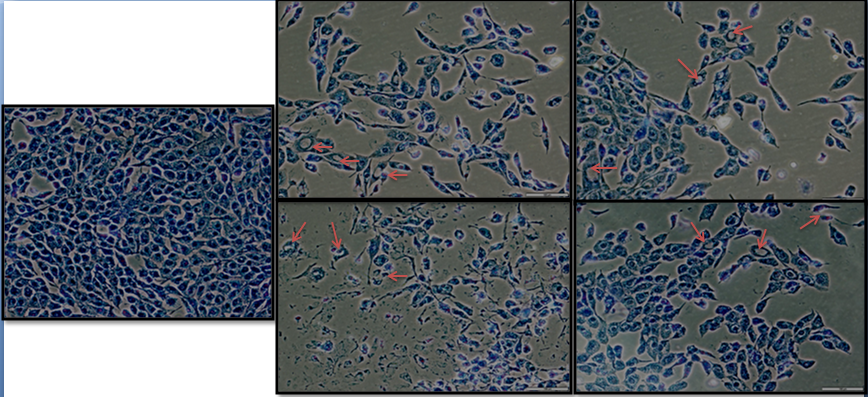

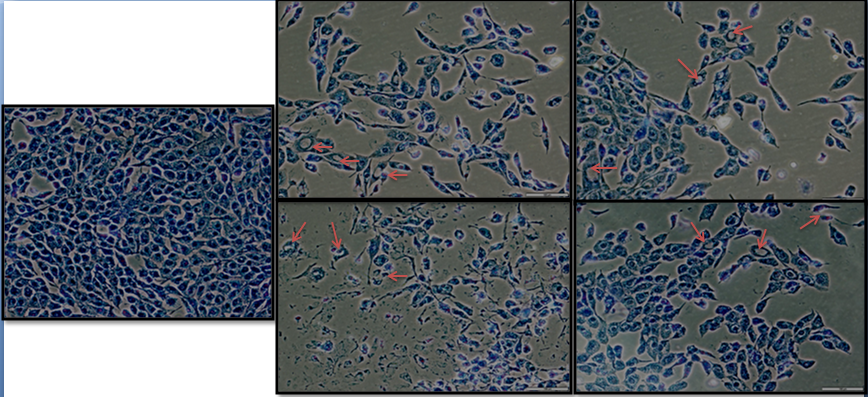


**Figure S1.** The anti-proliferation effect of camptothecin on MCF-7 cells. (A) Cells were treated with camptothecin at the indicated concentration for 3 days. Data represent three independent experiments performed in triplicate. Asterisks denote differences with statistical significances compared to untreated cells (** represents *P*<0.005). *P*-values were obtained from a two-tailed *t* test. (B) Cells were stained with methylene blue and observed under microscope with 40x magnification. (i) Untreated cells, (ii) Cells were treated with 0.35 µg/mL camptothecin for 3 days. Red arrows indicate cells with chromatin condensation at the nuclear periphery.

**

**

**

**(B)**

**(A)**
